# Imatinib-induced liver cirrhosis in a patient with advanced gastrointestinal stroma tumor (GIST)

**DOI:** 10.1186/1471-2407-12-186

**Published:** 2012-05-21

**Authors:** Christoph Seidel, Martin Fenner, Florian Länger, Heike Bantel, Arnold Ganser, Viktor Grünwald

**Affiliations:** 1 Department of Hematology, Hemostasis, Oncology and Stem cell transplantation, Hannover Medical School, Carl-Neuberg Strasse 1, 30625, Hannover, Germany; 2 Department of Gastroenterology, Hepatology and Endocrinology, Hannover Medical School, Carl-Neuberg Strasse 1, 30625, Hannover, Germany; 3 Institute of Pathology, Hannover Medical School, Oncology and Stem cell transplantation,, Carl-Neuberg Strasse 1, 30625, Hannover, Germany

**Keywords:** GIST, Imatinib, Liver cirrhosis

## Abstract

**Background:**

The use of imatinib mesylate is associated with a progression free survival of 41 months in first line treatment of metastatic or locally advanced gastrointestinal stromal tumors (GIST) and other studies approved that adjuvant imatinib treatment improves the recurrence-free survival in patients with GIST. Current recommendations include 1 year adjuvant treatment in GIST patients at risk but active studies explore different durations of treatment with an interval of up to 5 years. While the most frequent adverse events (AEs) are blood count alterations, abdominal discomfort and edema, the occurrence of grade 3 or 4 increase of AST or ALT is specified with 2.1% and 2.7% respectively.

**Case presentation:**

We report a 49-year old male with a gastrointestinal stromal tumor (GIST) of the small bowel who developed liver cirrhosis under adjuvant imatinib treatment.

**Conclusions:**

Our report supports the notion that imatinib-induced hepatotoxicity may lead to acute liver damage with subsequent cirrhotic remodelling. Patients developing grade 3 or 4 hepatotoxicity during imatinib treatment should therefore be carefully evaluated for chronic liver disease.

## Background

The selective tyrosine kinase inhibitor imatinib mesylate has dramatically influenced the treatment of chronic myeloid leukemia (CML) and gastrointestinal stromal tumors (GIST). First line treatment of metastatic or locally advanced GIST with imatinib is currently associated with a PFS of 41 months [1]. The basis for imatinib in the treatment of GIST has been further broadened by other studies, which showed that adjuvant treatment with imatinib improves the recurrence-free survival in patients with GIST [2,3]. As imatinib treatment is commonly well tolerated, the most frequent adverse events (AEs) are blood count alterations, abdominal discomfort and edema. The adjuvant Z9100 GIST trial reported on grade 3 or 4 increase of AST or ALT in 2.1% and 2.7% respectively, indicating the rather rare occurrence of hepatotoxicity [3]. With the implementation of imatinib for adjuvant treatment in GIST more patients are exposed to imatinib, which may also increase the occurrence of imatinib-induced liver damage. Current recommendations include 1 year adjuvant treatment in GIST patients at risk, but active studies explore different durations of treatment with an interval of up to 5 years duration. We report on a case with liver cirrhosis in a patient with advanced GIST and high-risk of recurrence during adjuvant treatment with imatinib.

## Case presentation

**Figure 1:**
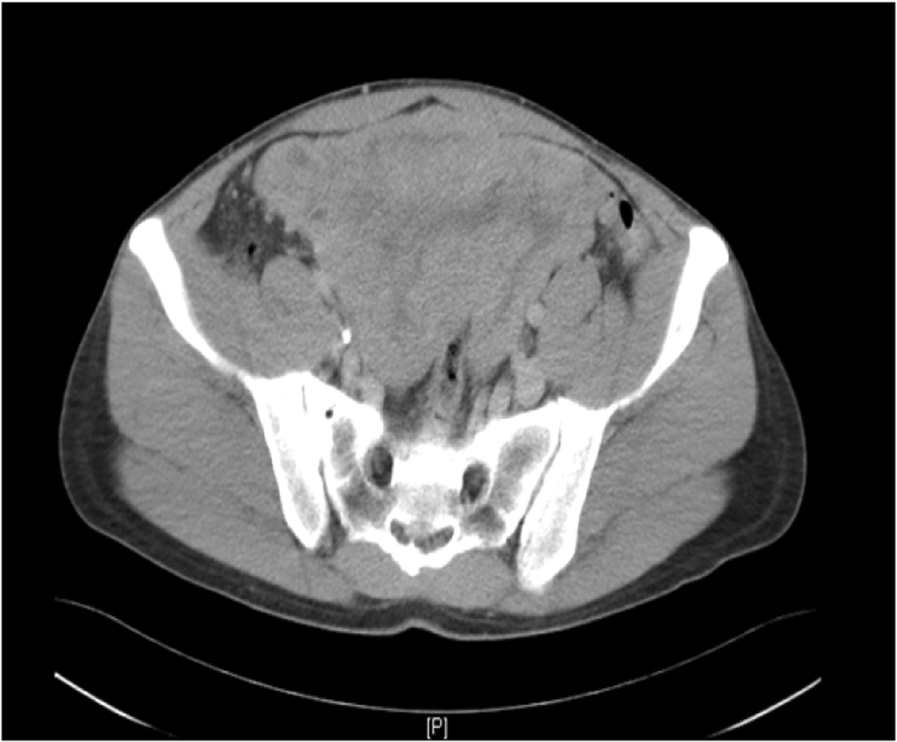
**CT scan of the patient.** GIST of the small bowel 18 x 10 cm in size when it was first diagnosed prior nedoadjuvant treatment.

**Figure 2:**
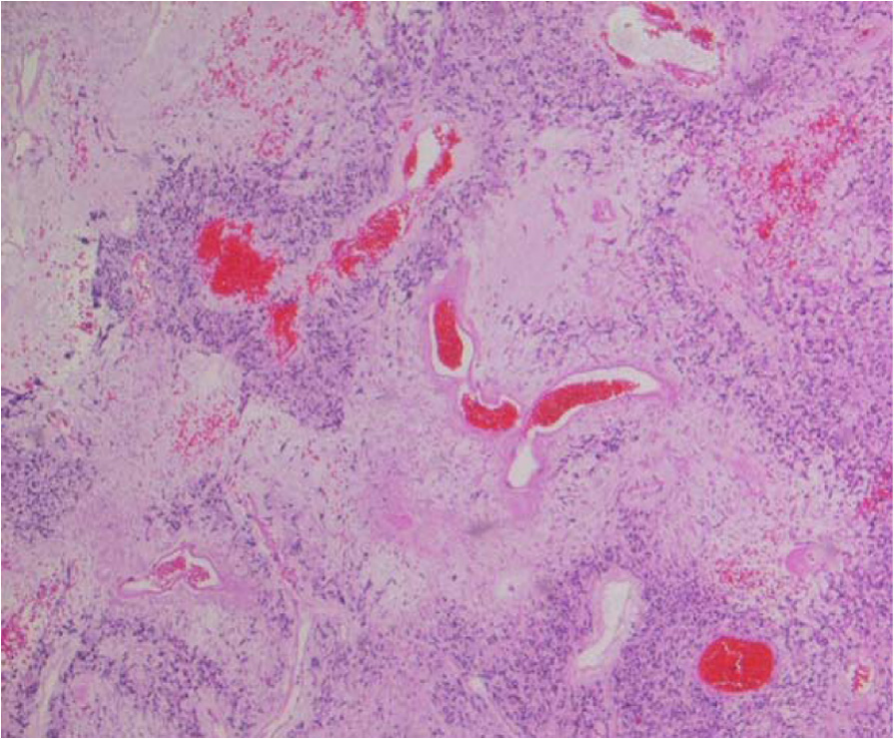
**Photomicrograph of the GIST, (Hematoxylin– eosin; original magnification, x200.** Tumor regression of 20% under neo-adjuvant treatment.

**Figure 3:**
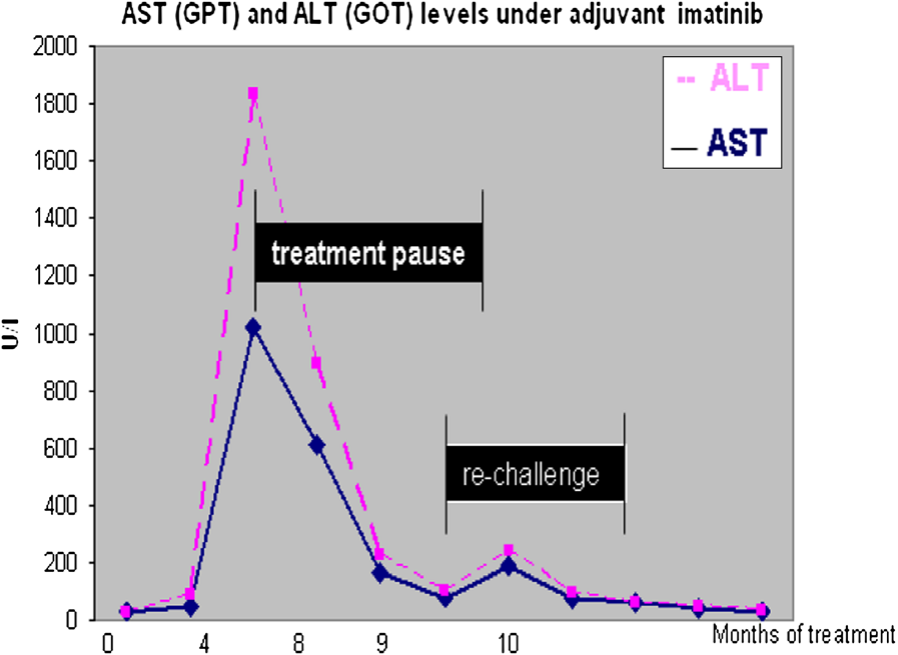
Development of transaminases under adjuvant imatinib treatment.

**Figure 4:**
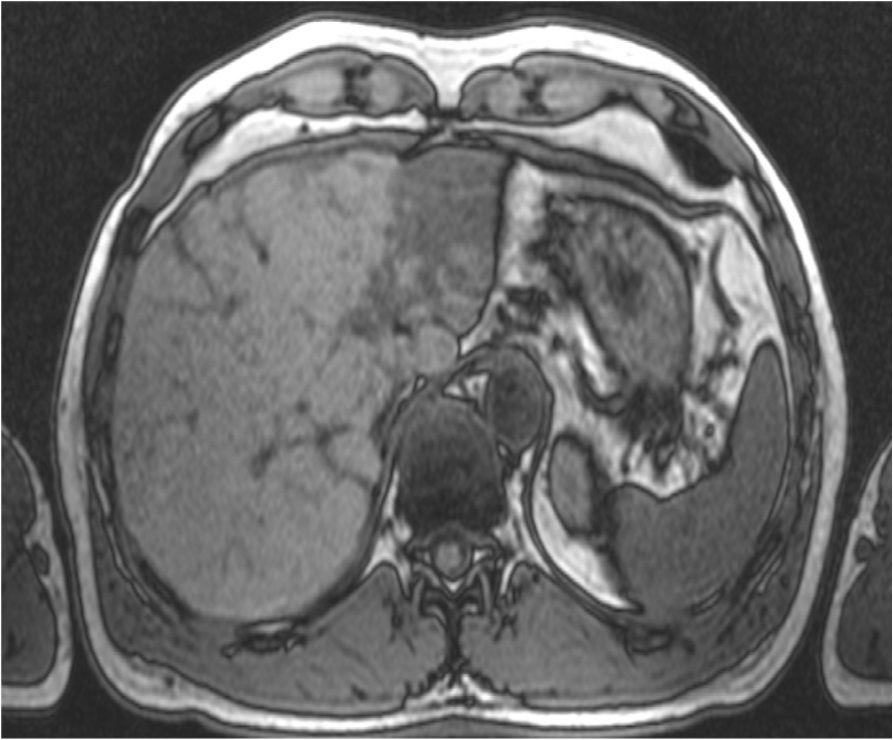
**MRI of the liver.** One month after discontinuing adjuvant treatment MRI shows clearly cirrhotic changes of the parenchyma.

A 49-year old male patient presented with flank pain and hematuria. The CT scan showed a large pelvic tumor 18 cm in diameter (Figure 1). A biopsy was performed and the pathologic examination revealed a spindle cell tumor with co-expression of CD117 and S100 protein, as well as a point mutation in c-kit exon 11, consistent with the diagnosis GIST. Due to the large tumor size, neoadjuvant therapy with imatinib 400 mg daily was initiated. After 6 months of treatment, the patient developed grade 3 hepatotoxicity with ALT and AST elevations from normal values to 882 U/l and 383 U/l, and an elevated bilirubin of 25 umol/l. Imatinib was paused for two weeks and parameters rapidly declined to normal limits. Treatment was then continued at 400 mg OD and sustained tumor shrinkage was seen in subsequent CT scans. After 16 months of neoadjuvant therapy tumor shrinkage ceased and tumor size was reduced from 18 cm to 14 cm (22% reduction). A complete tumor resection was performed at that time. Consistent with the clinical response, the pathological examination revealed a tumor regression of 20% (Figure 2) with low expression of proliferation markers (MIB <1%). Due to the high risk of recurrence, adjuvant therapy with imatinib 400 mg OD was initiated. After 7 months of adjuvant treatment, grade 4 hepatotoxicity developed with an ALT 1837 U/l and AST 1022 U/l (Figure 3) and an elevated bilirubin of 90 umol/l. Imatinib was paused for a total duration of 8 weeks, and subsequently the elevated transaminases declined to values within normal limits. Subsequent re-challenge of imatinib was associated with another episode of hepatotoxicity after 14 days of re-exposure. A corresponding MRI showed the development of cirrhotic changes of the liver (Figure 4), which had been absent at the previous MRI 3 months before. Other possible causes of liver damage, including metastasis, alcoholic liver disease, hepatitis A, B, C, and E, autoimmune hepatitis and metabolic disorders were excluded. Imatinib was discontinued permanently and the transaminases returned to normal values within 6 months. Fifteen months after discontinuation of imatinib the patient is still without relapse, but cirrhotic changes of the liver remained sustained on MRI.

## Discussion

To our knowledge this is the first case, which reports on the development of liver cirrhosis during adjuvant imatinib treatment in a patient with GIST. Hepatotoxicity has been observed in about 2–5% of patients receiving imatinib [4-6]. Most of them present with mild temporary liver enzyme elevations, which resolve after dose reductions or discontinuation of imatinib treatment [7,8]. However, acute liver failure has also been reported sporadically during imatinib treatment [9-11]. Histologic changes have been described in some of these cases and consist of inflammation, fatty degeneration, or necrosis of the liver [10,12,13]. Due to the transient nature of toxicity, re-exposure to imatinib is warranted in most cases [14]. Prospective studies in patients with pre-existing liver disease showed that imatinib can be administered safely in these patients, indicating a different mechanism for hepatic toxicity [15,16]. However, late changes have been recognized in a patient with CML who was diagnosed with liver cirrhosis one year after cessation of treatment due to imatinib-induced acute liver failure [14]. Therefore a close follow up should be mandatory. In asymptomatic patients constant liver enzyme measurements should be adequate. If cirrhotic changes already occurred, liver enzyme quantifications every 3 months with ultrasound of the abdomen and Alpha-Fatal Protein measurements twice a year are performed at our institution. If signs of consecutive liver damage without evident morphological changes of the parenchyma occur, transient elastography could represent an elegant screening method [17].

As we move forward to adjuvant therapy in GIST, a much larger proportion of patients will receive drug exposure and currently the ideal duration of adjuvant imatinib treatment in high risk GIST remains undefined. The work of Dematteo et al. indicates that the recurrence free survival seems to adapt after a treatment free interval of 18 months. To determine the ideal period of adjuvant imatinib treatment prospectively, clinical trials are conducted to test the efficacy of a prolonged exposure to adjuvant imatinib treatment in GIST for 5 years (NCT00867113) or to evaluate the effects of 12 months versus 36 months (NCT00116935).

## Conclusion

Our report supports the notion that chronic liver disease can be a consequence of imatinib-induced hepatotoxicity. In consequence a careful follow-up strategy should be implemented for patients with grade 3 or 4 hepatic toxicity.

## Consent

Written informed consent was obtained from the patient for publication of this case report and any accompanying images. A copy of the written consent is available for review by the Editor-in-Chief of this journal.

## Competing interests

The authors declare that they have no competing interests.

## Authors’ contribution

CS: reviewed the literature, drafted and edited the manuscript; MF: drafted and edited the manuscript; FL: processed and provided pathology; HB: Helped in editing the manuscript; gave advice in terms of gastroenterologic problems; AG: drafted and edited the manuscript; VG: reviewed the literature, drafted and edited the manuscript All authors were involved in the patient active management. All authors read and approved the final manuscript.

## Pre-publication history

The pre-publication history for this paper can be accessed here:

http://www.biomedcentral.com/1471-2407/12/186/prepub
